# Relative distribution of HPV genotypes in histological cervical samples and associated grade lesion in a women population over the last 16 years in Burgundy, France

**DOI:** 10.3389/fmed.2023.1224400

**Published:** 2023-08-11

**Authors:** Christelle Auvray, Serge Douvier, Odile Caritey, Jean-Baptiste Bour, Catherine Manoha

**Affiliations:** ^1^Department of Microbiology, Virology Laboratory, Dijon University Hospital, Dijon, France; ^2^Department of Gynecology and Obstetrics, Dijon University Hospital, Dijon, France

**Keywords:** human papillomavirus, genotypes, relative distribution, fluctuation, histological samples, emergence, HPV52

## Abstract

Human papillomavirus is a predominant sexually transmitted viral pathogen. Our objective was to analyze the relative distribution of genotypes over time and to determine the genotypes associated with adverse clinical lesions. The study was based on data from adult women with cytological abnormalities from whom histological samples were obtained from 2005 to 2021. HPV genotyping was performed using PCR and INNO-LiPA assay (Fujirebio). Among the 1,017 HPV-positive biopsies, 732 (72%) were infected with a single HPV genotype and 285 (28%) were infected with several HPV genotypes. Most of the infections involved the high-risk genotypes 16, 31, and 52. Throughout the study period, HPV 16 was the most encountered genotype (541, 53.2%), while HPV 18 was rather under-represented (46, 4.5%), especially in invasive cervical carcinoma. HVP52 (165, 16.2%) was detected mainly from 2008 to 2014, and its distribution reached 19.7% in 2011. Such epidemiological data underlines the possibility of an emergence of a high-risk genotype. The most detected low-risk HPV in combination with high-risk HPV was HPV 54 in 6.5% of samples. Monoinfection by HPV 16 led statistically more often to severe lesions than multi-infection involving HPV 16 (*p* < 0.001), while for HPV 52, 31 or 33, multi-infections were significantly associated with severe lesions (*p* < 0.001 for each of these three genotypes). HPV 16 was involved in 55.2% of high-grade lesions and *in situ* carcinoma and 76.3% of invasive carcinomas. In severe lesions, HPV 16 participation was predominant, whereas diverse genotypes were seen in low-grade lesions. Importantly, we observed that high-risk genotypes, for example HPV 52, can emerge for a few years then decrease even without vaccine pressure.

## Introduction

The human papillomavirus (HPV), which belongs to the *Papillomaviridae* family, is a pathogen that infects the epithelial tissue. It is the main risk factor for cervical cancer and is thus highly clinically relevant (1). HPV is mainly transmitted through sexual contact, and most people are infected with HPV shortly after the onset of sexual activity. HPV can also lead to anogenital cancers affecting the anus, vulva, vagina, and penis, as well as to oropharyngeal cancers located in the pharynx, larynx and oral cavity. Anogenital and oropharyngeal cancers linked to HPV occur in both men and women (2, 3). The high-risk (HR) HPV genotypes are responsible for severe lesions, and the persistence of oncogenic HR HPV infection is the main cause of cervical cancer. The DNA integration of HPV into the infected cell genome contributes to malignant transformation of host cells (4, 5). Some cofactors (viral, immune response of the host, or host behavior) may also contribute to the development of cervical cancer. However, the specific role of these cofactors in the persistence and progression of cervical infections due to HPV is not well known. HPV 16 and HPV 18 have been identified as the most prevalent HPV types, and they are associated with more than 70% of cervical cancers and are accountable for 50% of precancerous cervical lesions (6, 7). Papillomavirus infections are common in sexually active women. Most infections clear spontaneously and have no clinical signs. Half of new HPV infections are undetectable after 6 to 12 months and more than 90% are undetectable at 3 years (8). Persistent HPV infection has been associated with cervical intraepithelial neoplasia (CIN) and cervical cancer. The natural history of HPV-induced cervical cancer is usually slow, and it remains unclear why only a fraction of HPV cases progress to cancer. Multi-infections with several HPV genotypes are frequent, but the frequency varies depending on the country, and the clinical impact is controversial (9–11).

In 2018, there were nearly 3,000 new cases of invasive cervical cancer in France and more than 1,000 deaths (12). Moreover, although the incidence and mortality of cervical cancer have declined over the past decades, this reduction has been slower since 2005 (13). Given the abundant diversity of HPV genotypes, we conducted an epidemiological investigation to better understand their relative distribution in histological samples to provide useful information for HPV vaccination programs. We analyzed samples collected over a 16-year period and explored the HPV genotypes found in mono- and multi-infections, the preferential links among them, and their association with clinical conditions.

A vaccination program was launched in France in 2007 with the quadrivalent Gardasil vaccine that targets HPV 6, 11, 16, and 18. In December 2019, a new vaccine, Gardasil 9, was implemented, targeting HPV 6, 11, 16, 18, 31, 33, 45, 52, and 58. The low-risk (LR) genotypes, HPV 6 and 11, are responsible for 90% of condyloma acuminate cases. The HR genotypes targeted by the Gardasil 9 vaccine are involved in 80% of CIN2+ lesions and 90% of cervical cancers. Unfortunately, the poor perception of the HPV vaccine has hampered its implementation in France (14, 15), and the coverage of the French population is among the lowest in Europe. A better understanding of the genotypic spectrum of HPV could inform preventative strategies against cervical cancer and help to measure of the impact of vaccination.

## Methods

### Specimen collection

This retrospective cohort study was conducted at the Dijon University Hospital, France. The samples were collected for diagnostic purposes in the Medical Gynecology department, from September 2005 to December 2021. No additional samples were collected for the purpose of this study. Patient non-opposition was confirmed. All the women had cervical abnormalities, at least a pap smear with LSIL. Samples were mainly conizations, cervical, endocervical or endometrial biopsies, as well as some paraffin blocks from the pathological anatomy laboratory. All samples were placed in tubes with a transport medium. Briefly, biopsies were dilacerated with a scalpel, paraffin embedded samples were shaved to obtain slices, and all samples were incubated in a proteinase K solution at 56°C with stirring for at least 2 h up to 16 h maximum. Proteinase K was inactivated at 95°C for 10 min. At this stage, samples could be frozen at −20°C prior to analysis.

Classification of lesions was as follows: group 0, included samples close to normal; group 1, called low-grade lesions, included cervical intraepithelial neoplasia (CIN)1 and flat condyloma; group 2, named high-grade lesions group, included CIN2, CIN3, adenocarcinoma *in situ* (AIS), and squamous cell carcinoma (SCC); and a third group included invasive cervical carcinoma (ICC).

### Genotyping

HPV infection was detected using, successively, two versions of the INNO-LiPA HPV Genotyping (Fujirebio) that can determine the presence of the 28 genotypes (version EXTRA from 2005 to 2014) or 32 genotypes (version EXTRA II from 2015 to 2021). The INNO-LiPA HPV Genotyping is a line probe assay for *in vitro* diagnostic use. It was designed for the identification of HPV genotypes by detection of specific sequences in the L1 region of the HPV amplification products that are subsequently hybridized using a single typing strip on which sequence-specific DNA probe lines and control lines are fixed. Classification of genotypes were indicated in the manufacturer’s instructions, according to Munoz et al. (16) and IARC Monographs volume 100B (17):


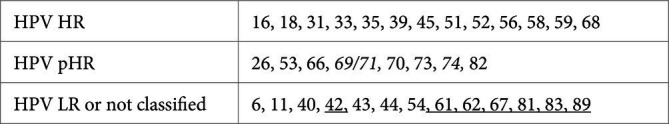


HR means high-risk, LR means low-risk, and p, probable. The underlined genotypes were detected only in the EXTRA II version, while those in italic were no longer detected. When HPV 52 and a weak signal for HPV 31 were detected, only 52 was recorded seeing as non-specific activity was attributed to HPV 31, as indicated by the manufacturer.

### Statistical analysis

Continuous variables were expressed as means ± standard deviation (SD), categorical variables were expressed as percentages, and 95% confidence intervals (CI) were indicated. Categorical variables were compared using the Chi-square test and continuous variables were compared using one-way analysis test or Kruskal–Wallis test, as appropriate. A value of *p* <0.05 was considered statistically significant. STATA v15 (StataCorp LLC, College Station, TX, United States) was used for the statistical analyses.

## Results

We analyzed the relative distribution of genotypes in histological specimens collected over a 16-year period, from 2005 to 2021. The samples (*n* = 1,017) were collected from female patients who were HPV-positive and analyzed using the INNO-LiPA hybridization test. Women were aged 18 to 92 (mean 38.9 years; median 37.5 years). Samples were obtained in the cervix (998, 98.1%), endometrium (2, 0.2%), vagina (3, 0.3%), vulva (9, 0.9%), or anus (5, 0.5%). As expected for this cohort, most of the women had high-grade lesions. Women with an HR HPV infection represented 97.8% (96.7–98.6%) of patients (*n* = 995), while only 29 women 2.9% (1.9–4.1%) were found to have either only a LR (*n* = 22) or an indeterminate genotype (*n* = 7). Moreover, 10 of these 29 samples were extra-cervical.

**Figure 1:**
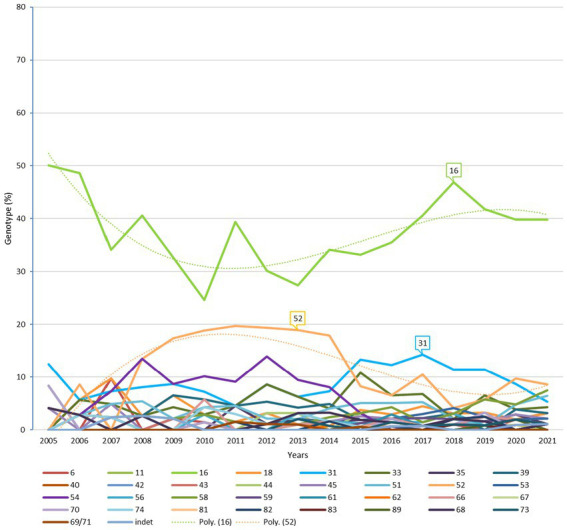
Relative distribution and trends of HPV genotypes (2005–2021). Dotted lines indicate polynomial regression curve for HPV 16 (green curve) and HPV 52 fluctuating genotype (orange curve).

The predominant genotypes were HR HPV 16, 52, and 31, followed by HR HPV 33 and LR HPV 54 (Figure 1). The most prevalent genotype was HPV 16 (541, 53.2%), followed by HPV 52 (165, 16.2%), and HPV 31 (142, 14/%); HPV 18 was rather under-represented throughout the study (46, mean 4.5%) (Figure 1). These four genotypes are all classified as high-risk. The distribution of the other genotypes targeted by Gardasil 9 (HPV 6, 11, 18, 45) was below 5%. Supplementary Figure S1 illustrates the distribution of genotypes included in the new Gardasil 9 vaccine (Supplementary Figure S1).

The distribution of HPV 16 was high (mean per year, 37.6% ± 7.1%) with a tendency to decrease over the years 2007–2016 when HPV 52 emerged (polynomial tendency curve 16 on Figure 1). HPV 52 was rarely detected in 2005 to 2007. It then emerged from 2008 to reach a distribution of 18.9% ± 2% from 2010 to 2014, and decreased to 7.6% ± 2.3% from 2015 to 2021 (polynomial tendency curve 52 on Figure 1).

Similar to HPV 52, the distribution of LR HPV 54 reached 10.4% ± 2.4% from 2008 to 2014, then decreased from 2015 to 2021 to 1% ± 1.1%.The distribution of HR HPV 31 was 12.6% ± 1.2% from 2015 to 2019 vs. 7.5% ± 2.1% for the rest of the study period. These genotypes (in descending order: 16, 52, 31, 33, 54, and 51) constituted 67.3% of the genotype cases (Figure 1).

**Figure 2:**
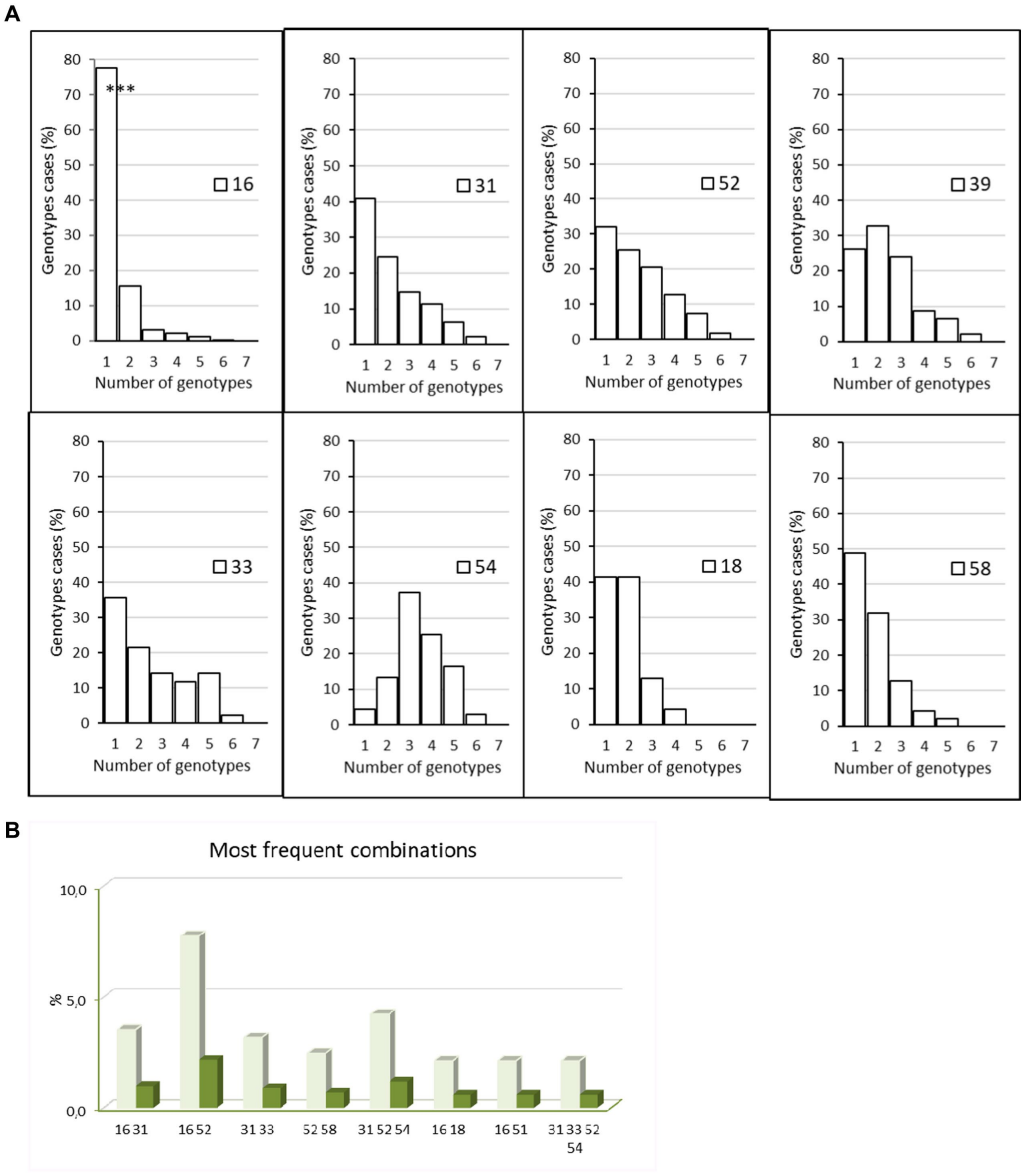
Mono infections and multi infections. **(A)** Percentage of women with HPV 16, 18, 31, 33, 39, 52, 54, and 58 with mono or multi-infections with 1–5 other genotype during the study period 2005–2021, *p* < 0.001 for HPV 16 (mono- vs. multi-infections), Chi-square test. **(B)** Most frequent combinations detected in patients during the study period, 2012–2021. Dark green: percentage of all infected patients, light green: percentage of multi-infected patients. The graphic highlights the dual infection by HPV 16 and 52.

Almost three quarters of samples revealed a monoinfection [732, 72% (69.1–74.7%)], including seven positive samples with indeterminate genotypes, and the remaining 285 samples were multi-infected [28% (25.3%–30.9%)]. When multi-infected (*n* = 285), women had an infection with 2 to 6 different HPV genotypes, dual infections were the most frequent (178, 62.5% of multi-infections), 61 samples included three different genotypes, 27 included four genotypes, 16 included five genotypes, and three samples had six genotypes, which led to the determination of 1,477 genotype results in total. HPV 16 was most frequently found as a single genotype (77.8% of HPV 16) whereas other HR types of HPV were often detected as coinfecting viruses (Figure 2A). The prevalence of HPV 16 mono-infection was far higher than HPV 16 in multi-infection: 77.8% (74.1%–81.2%) vs. 27.1% (23.3%–31.3%). HPV 31, 33, 39, 52, and 54 were often associated with other genotypes, and HPV 18 was detected mainly alone or in dual infections (Figure 2A). The most frequently observed combinations were dual infections with HPV 16 and 52 (in 7.7% of coinfected patients) followed by the combination of HPV 31, 32, 54 (in 4.2% of coinfected patients) (Figure 2B).

Most coinfections involving HPV 16 were dual infections (84/120, 70%). Of these, only eight involved an LR HPV as a coinfectant (9.5%). We then focused on preferential associations for prevalent genotypes (Supplementary Table S1). For the HR genotypes 18, 58, and 68, a quarter or more of coinfections occurred as follows: HPV18 coinfected with HPV 39 or 16 (36.8% and 23.7% of coinfection cases, respectively), HPV 58 with HPV16 or 52 (27% and 29.7%), and HPV 68 with HPV 39 (25.6%). When coinfected, HPV 16 was most often found with HPV 52 (22% of cases). Interestingly, HPV 33 was infrequently co-infected with the prevalent HPV 16 genotype (7.9%). As expected, low-risk genotypes were poorly represented in this cohort. Only HPV 54 was frequently encountered in association with HPV 52 and 31 (29.9% and 26.2% of cases).

**Figure 3:**
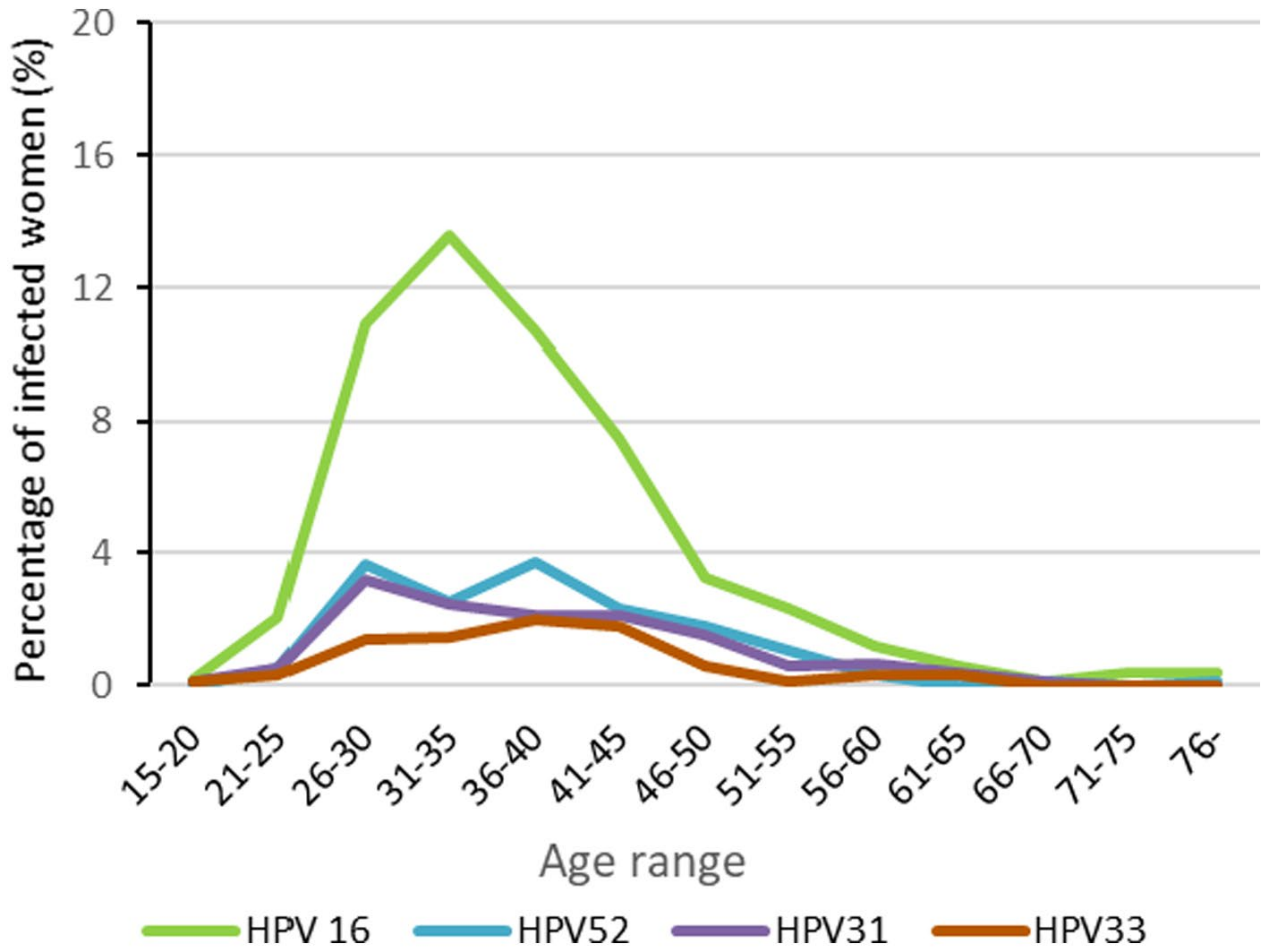
HPV distribution of the most prevelent genotypes by age range results are expressed as percentages of total number of womens.

The mean age of the cohort was 38.9 years. The mean age of patients with monoinfection was 39.3 years while multi-infected women had a mean age of 37.9 years (*p* < 0.05). HPV 16 was most frequent in women 26–45 years (*p* < 0.001). Although not statistically significant, the distribution of HPV 52, 31, and 33 also appeared to be higher from 26 to 45 years old (Figure 3).

**Figure 4:**
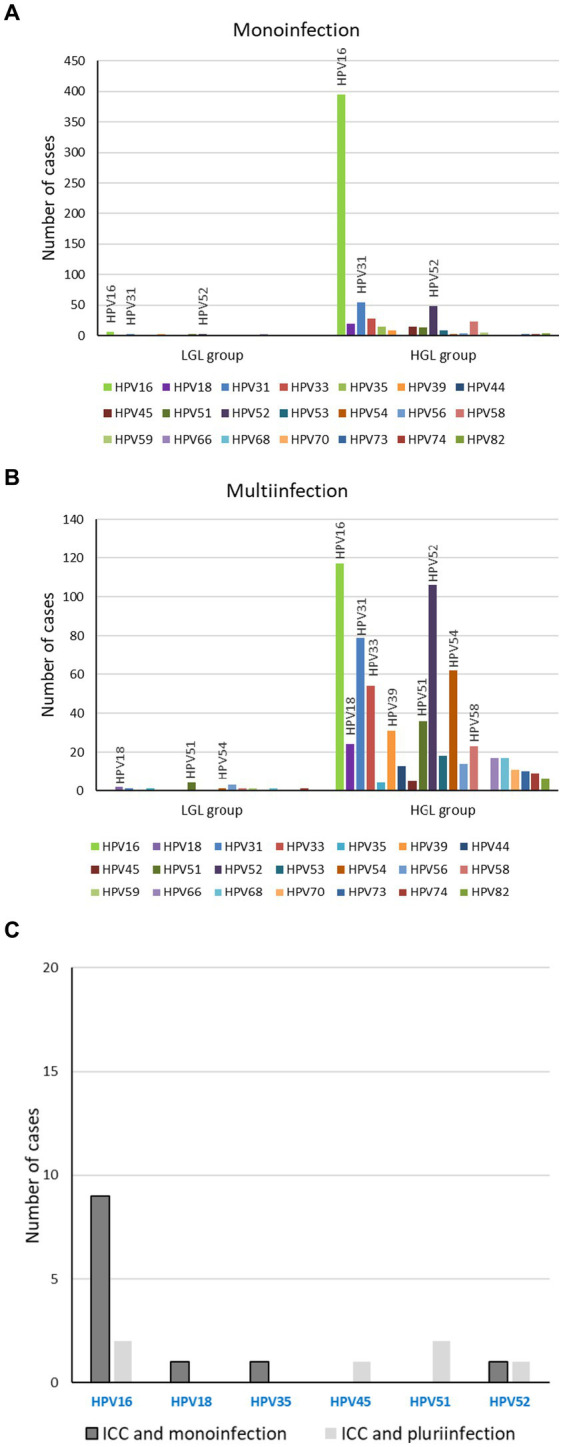
Clinical pathology associated to histrorical samples according to genotype. Genotypes associated to group 2 of lesions, low-grade lesions and high-grade lesions, monoinfection **(A)** or multi-infection **(B)**; Genotypes associated to group 3 of lesions, ICC, monoinfection and multi-infection **(C)**.

HPV 16 was the most frequent genotype in all histological grades, and its frequency increased with the severity of the lesions. Specifically, 13.5% (5.6%–25.8%) of low-grade lesions were HPV-16-positive, 55.2% (51.9%–58.5%) of high-grade lesions and 76.3% (59.6%–88.6%) of ICC (*p* < 0.001) (Figure 4). HPV 52 and 31 were detected, respectively, in 16.9% and 14.7% of the high-grade lesions. Depending on the genotype involved, we found that either the monoinfections or the multi-infections were associated with severe lesions. HPV 16 monoinfections were associated with severe lesions while multi-infections including genotypes 31, 33 and the emergent HPV 52 genotype were associated with severe lesions.

We also explored the age of women with single-type HPV 16 infections in the different lesion classification groups. Women with HPV 16 in group 3 (mean 44.6 years old) were older than those in group 2 (mean 37.3 years old) while in group 1 (low-grade lesions), the mean age was 33.0 years old (*p* < 001). Associations between age and any other genotypes could not be calculated because of the large diversity of genotypes in group 1 and the limited number of cases in group 3.

## Discussion

HPV infection is associated with the development of several types of cancer, and it is therefore necessary to reduce the incidence of infections of all prevalent HR genotypes. Genotype distribution and variation in severe lesions needs to be better understood, and this information can be used to design vaccine strategies and evaluate their impact. We found single type infection to be most common, and HPV 16 was shown to predominate over the 16 years of the study, from 2005 to 2021, for both the distribution and the associated disease severity. On the contrary, HPV 52 emerged as the second most common genotype over a period of several years and then decreased. HPV 16 was defined as the HPV genotype with the highest risk of causing cancer of the cervix and several other sites (18), and HPV 18 was found to be the second most prevalent HR HPV in cervical cancer in 2003 (16). HPV 18 remained relatively infrequent throughout the study at around 4.5%. This is in accordance with a study performed in France and based on a previous period from 1999 to 2005, during which HPV 16 was by far the most common HPV type associated to CIN 2/3 while HPV 18 was observed only in 4% of the CIN 2/3 samples (19). However, worldwide, HPV 18 positivity in biopsies was reported to reach 8.9% in CIN2/3 from 1999 to 2011 (20). The proportion of HPV 18 was also found to be 16 to 19% in ICC (20, 21). Surprisingly, we report here a very low level of HPV 18 in ICC, which is potentially due to the study period or local prevalence.

The distribution of genotypes can vary depending on the country (1), and we show here that it is also variable over time in the same location. We reported in particular the emergence and then the decline in HPV 52 over time. HPV 16 as well as HPV 52 and 33 are of public health concern and are included in the nonavalent vaccine. We found a relatively large proportion of HPV 52 in the biopsies: it rose rapidly to 13% in 2008, stabilized at about 19% for six years, and then declined and remained around 8% the following years. During the same period, in 2010–2012, endocervical cytology samples from Mexico were found to have a high prevalence of HPV 51 and 52 (around 37%), even exceeding HPV 16 (22). A meta-analysis based on studies from 1994 and 2012 reported that the prevalence of HPV 52 in CIN2/3 samples was high, at about 16.5% in Asia and about 11.2% in the Americas and Europe (23). Although we found that the distribution of HPV 52 decreased to 8% after 2015, HPV 52 was still common in 2017 in China (20%) among women with cervical precancerous lesions (24). HPV genotype distributions vary between different regions as underlined elsewhere, but also locally over time as shown here. This can be explained by the regional dominance of variants. In Asia, the B2 sublineage of HPV 52 is greatly dominant and frequently detected in serious cervical lesions compared to the HPV 52 a lineage, which predominates in Europe (25). Moreover, some variants of HPV 52 display specific HPV 52 mutations that contribute to lesion severity (26, 27). The geographical and chronological variability in the prevalence of HR human papillomavirus genotypes in cervical intraepithelial neoplasia lesions underlines the importance of continuously monitoring HPV genotype prevalence within a country in order to accurately assess the efficacy of HPV vaccines.

From 2007, two vaccines against the papillomavirus were available, Cervarix, which targets HPV 16 and 18 but is no longer recommended, and Gardasil, which targets HPV 6, 11, 16, and 18. Recently, virus–like particles (VLPs) to protect against HPV 31, 33, 45, 52, and 58 were added to these four genotypes to produce the updated Gardasil 9 vaccine. Gardasil 9 was implemented in December 2019 in France, when the women included in our study were too old to be eligible for vaccination. Therefore, the observed emergence and decline of HPV 52 cannot be attributed to any vaccine pressure against this genotype. It is worth noting, however, that vaccination may facilitate an increase in HR HPV types not targeted by the vaccine (28). Rather frequently, genotypes such as 31, 33, 52, and 54 were found to be part of multi-infections. Regarding the other genotypes included in Gardasil 9, HPV 18 was associated preferentially with HPV 39, while HPV 58 was associated preferentially with HPV 52; HPV 45 was infrequent. We also found a preferential association between HPV 16 and 52. An antagonism by viral interference has been proposed between HPV 16 and low-risk HPV 6/11 (29). Our data showed a low capacity of HPV 16 to co-infect with low-risk genotypes except HPV 54 when also combined with other HR HPVs. The interaction between multiple HPVs has been suspected to affect oncogenic risk, but the impact of multiple infections on the risk of cervical lesions has not been established yet. Whether these infections occur by chance or as a result of interactions between HPV genotypes is still unclear (30). Several studies have reported that multiple HPV infections were more closely related to high-grade lesions compared to single infections (11, 31). We add here that this assertion is true for several genotypes (HPV 31, 33, 52), except for HPV 16. In Italy, Iacobone et al. established that HPV multi-infections were significantly associated with lower risk of CIN2+, whereas single infections were more likely in cervical cancers and precancerous lesions (32). Other studies have also shown reduced high-grade squamous intraepithelial lesion (HSIL) rates for multiple HPV infections compared to single-genotype infection, with no additive or synergistic effect, suggestive of possible intergenotypic competition or more effective immune response triggered by multiple infections (33). Recently, HPV 16 was reported to have a lower risk of CIN 3+ when co-infected with other types than single HPV 16 infection (34). In our study, HPV 16 was by far the most dominant genotype and was found mainly as single HPV infection in severe lesions, underlying its pathogenic role.

Regarding only dual infections, ours results indicate that they were more likely to lead to severe lesions when they included HPV 16. Specific HPV dual-infections such as HPV 16–68 (35) or HPV 16–58 (31) seem particularly prone to increase risk of intraepithelial lesions and cervical cancer in women. However, here, these coinfections were very infrequent. A study in Italy showed that the most common co-infections in patients with CIN were HPV 16–18, 51–52, and 16–51–52 (36). In Brazil, the co-infection of HPV 16–18 was related to a higher future risk for both cervical adenocarcinoma *in situ* and ICC (37). In our study, the coinfection of HPV 16–52 was the most frequent. None of the samples with this combination were low-grade lesions – they were all classified as group 2 lesions. In two recent studies, HPV 52 was shown to be one of the five dominant HPV genotypes found in CIN 2/3 and cancer (23, 38). In particular, HPV 52 and 58 have been reported to be common types in Asia among CIN 2/3 and ICCs (23, 38), while we found a fairly low distribution of HPV 58 in severe histological lesions and a large but fluctuating prevalence of HPV 52.

Here, we found that the majority of ICC was associated with HPV 16 monoinfection (76.3%), in accordance with previous reports (63%–65%) (20, 39, 40). These results confirm that HPV 16 monoinfections confer an increased risk of developing high-grade lesions and ICC. For single infections with HPV 16, increased viral load and integrated viral genomes have been significantly associated with prevalent HSIL (41). Moreover, a threshold for HPV 16 viral load was associated with disease prognosis, whereas it could not be defined for HPV 18 (42–44). Among the ICC cases, we reported only one case involving HPV 18 that was in a dual infection with HPV 39. The genotypes, apart from HPV 16, most frequently associated with ICC are HPV 18 worldwide, HPV 45 in Africa and Latin and Central America, and HPV 58 in Asia (20). HPV 18 positivity was reported to vary very little between normal cytology and CIN3 (around 7.6%), but this level doubled in ICC (20). Moreover, since the prevalence of HPV 18 in SCC and ICC was higher at younger age, it has been suggested that HPV 18 induces cancer rapidly (45), whereas the risk of HPV 16 infection in cancer was not influenced by age (40, 45). Results on the associations between age, HPV 16 and high-grade lesions are conflicting. Correa et al. reported that the risk of HPV 16 infection in CIN3 cases increased with age while another recent study from Finland showed that HPV-16-type distribution in HSIL was more prevalent in women younger than 30 years (40, 46). In line with Correa et al., we did find a significant association between age, HPV 16 genotype and severe lesions in biopsy samples.

Cervical cancer accounts for 83% of HPV-attributable cancer, two-thirds of which occur in less developed countries. HPV-DNA integration into cellular chromatin is a necessary event contributing to carcinogenesis. The persistence over time of a HR HPV infection (47, 48) and the presence of cofactors increases the risk of occurrence of cervical cancer (49). Fortunately, genotypes covered by the nonavalent vaccine contributed to 85.2% of CIN2 lesions, 97.9% of CIN3 lesions, and 93.8% of cancers (50). Universal access to vaccination is the key to avoiding most cases of HPV-attributable cancer (1) and the French National Cancer Control Plan (NCCP) launched in 2014 fixed the objective to improve human papillomavirus vaccination coverage. However, Gardasil has failed to reach coverage goals due to a context of vaccination hesitancy in France (14, 51).

In conclusion, this study underscores the prevalence of HR HPV 16 over time in Burgundy in women undergoing biopsies or resections after abnormal colposcopies. The strong presence of HPV 16 has been consolidated over the years despite some vaccine pressure. In our population, which was not eligible for vaccination with Gardasil 9, we evidenced a spontaneous fluctuation of some genotypes over the 16 years. Such fluctuations could bias the results of vaccine policy evaluations.

## Data availability statement

The raw data supporting the conclusions of this article will be made available by the authors, without undue reservation.

## Ethics statement

Ethical review and approval was not required for the study on human participants in accordance with the local legislation and institutional requirements. Written informed consent from the participants was not required to participate in this study in accordance with the national legislation and the institutional requirements.

## Author contributions

J-BB, SD, and CM: study concept and design. SD, CA, J-BB, and CM: acquisition, analysis and interpretation of data. OC: laboratory detection. CM: writing. J-BB, SD, CA, and OC: critical revision. All authors contributed to the article and approved the submitted version.

## Conflict of interest

The authors declare that the research was conducted in the absence of any commercial or financial relationships that could be construed as a potential conflict of interest.

## Publisher’s note

All claims expressed in this article are solely those of the authors and do not necessarily represent those of their affiliated organizations, or those of the publisher, the editors and the reviewers. Any product that may be evaluated in this article, or claim that may be made by its manufacturer, is not guaranteed or endorsed by the publisher.

## Abbreviations

HPV: human papilloma virus
LR: low-risk
HR: high-risk
LGL: low-grade lesions
HGL: high-grade lesions
CIN: cervical intraepithelial neoplasia
ADC: adenocarcinoma
ISC: *In situ* carcinoma
SCC: squamous cell carcinoma
ICC: invasive cervical carcinoma

## References

[1] de Martel C Plummer M Vignat J Franceschi S. Worldwide burden of cancer attributable to HPV by site, country and HPV type. Int J Cancer. (2017) 141:664–70. doi: 10.1002/ijc.30716, PMID: 28369882 PMC5520228

[2] Sammarco ML Ucciferri C Tamburro M Falasca K Ripabelli G Vecchiet J. High prevalence of human papillomavirus type 58 in HIV infected men who have sex with men: a preliminary report in Central Italy. J Med Virol. (2016) 88:911–4. doi: 10.1002/jmv.24406, PMID: 26467111

[3] Sasidharanpillai S Ravishankar N Kamath V Bhat PV Bhatt P Arunkumar G. Prevalence of human papillomavirus (HPV) DNA among men with oropharyngeal and Anogenital cancers: a systematic review and Meta-analysis. Asian Pac J Cancer Prev. (2021) 22:1351–64. doi: 10.31557/APJCP.2021.22.5.1351, PMID: 34048162 PMC8408381

[4] Moody CA Laimins LA. Human papillomavirus oncoproteins: pathways to transformation. Nat Rev Cancer. (2010) 10:550–60. doi: 10.1038/nrc2886 20592731

[5] Williams VM Filippova M Soto U Duerksen-Hughes PJ. HPV-DNA integration and carcinogenesis: putative roles for inflammation and oxidative stress. Future Virol. (2011) 6:45–57. doi: 10.2217/fvl.10.73, PMID: 21318095 PMC3037184

[6] de Sanjose S Quint WG Alemany L Geraets DT Klaustermeier JE Lloveras B. Human papillomavirus genotype attribution in invasive cervical cancer: a retrospective cross-sectional worldwide study. Lancet Oncol. (2010) 11:1048–56. doi: 10.1016/S1470-2045(10)70230-8, PMID: 20952254

[7] WHO (2022). Available at: https://www.who.int/en/news-room/fact-sheets/detail/human-papillomavirus-(hpv)-and-cervical-cancer. (Accessed July 18, 2023)

[8] Rodriguez AC Schiffman M Herrero R Wacholder S Hildesheim A Castle PE. Rapid clearance of human papillomavirus and implications for clinical focus on persistent infections. J Natl Cancer Inst. (2008) 100:513–7. doi: 10.1093/jnci/djn044, PMID: 18364507 PMC3705579

[9] Mendez F Munoz N Posso H Molano M Moreno V van den Brule AJ. Cervical coinfection with human papillomavirus (HPV) types and possible implications for the prevention of cervical cancer by HPV vaccines. J Infect Dis. (2005) 192:1158–65. doi: 10.1086/444391, PMID: 16136457

[10] Spinillo A Dominoni M Boschi AC Sosso C Fiandrino G Cesari S. Clinical significance of the interaction between human papillomavirus (HPV) type 16 and other high-risk human papillomaviruses in women with cervical intraepithelial neoplasia (CIN) and invasive cervical Cancer. J Oncol. (2020) 2020:1–9. doi: 10.1155/2020/6508180 PMC7648694 33178274

[11] Kim J Kim M Park JY. Evaluation of the characteristics of multiple human papillomavirus (HPV) infections identified using the BD Onclarity HPV assay and comparison with those of single HPV infection. J Pathol Transl Med. (2022) 56:289–93. doi: 10.4132/jptm.2022.08.02, PMID: 36128865 PMC9510038

[12] Defossez G Le Guyader‑Peyrou S Uhry Z Grosclaude P Colonna M Dantony E. Estimations nationales de l’incidence et de la mortalité par cancer en France métropolitaine entre 1990 et 2018. Volume 1 – Tumeurs solides. Saint‑Maurice (Fra): Santé publique France (2019) 372.

[13] Arbyn M Raifu AO Weiderpass E Bray F Anttila A. Trends of cervical cancer mortality in the member states of the European Union. Eur J Cancer. (2009) 45:2640–8. doi: 10.1016/j.ejca.2009.07.018, PMID: 19695864

[14] Heard I Tondeur L Arowas L Demazoin M Falguieres M Parent Du Chatelet I. Effectiveness of human papillomavirus vaccination on prevalence of vaccine genotypes in young sexually active women in France. J Infect Dis. (2017) 215:757–63. doi: 10.1093/infdis/jiw639, PMID: 28011911

[15] Ohannessian R Constantinou P Chauvin F. Health policy analysis of the non-implementation of HPV vaccination coverage in the pay for performance scheme in France. Eur J Pub Health. (2019) 29:23–7. doi: 10.1093/eurpub/cky173, PMID: 30252035

[16] Munoz N Bosch FX de Sanjose S Herrero R Castellsague X Shah KV. Epidemiologic classification of human papillomavirus types associated with cervical cancer. N Engl J Med. (2003) 348:518–27. doi: 10.1056/NEJMoa021641 12571259

[17] IARC Working Group on the Evaluation of Carcinogenic Risks to Humans. Biological agents. IARC Monogr Eval Carcinog Risks Hum. (2012) 100:1–441. PMID: 23189750 PMC4781184

[18] Bouvard V Baan R Straif K Grosse Y Secretan B El Ghissassi F. A review of human carcinogens—part B: biological agents. Lancet Oncol. (2009) 10:321–2. doi: 10.1016/S1470-2045(09)70096-8, PMID: 19350698

[19] Pretet JL Jacquard AC Carcopino X Monnier-Benoit S Averous G Soubeyrand B. Human papillomavirus genotype distribution in high grade cervical lesions (CIN 2/3) in France: EDITH study. Int J Cancer. (2008) 122:424–7. doi: 10.1002/ijc.23093 17893883

[20] Guan P Howell-Jones R Li N Bruni L de Sanjose S Franceschi S. Human papillomavirus types in 115,789 HPV-positive women: a meta-analysis from cervical infection to cancer. Int J Cancer. (2012) 131:2349–59. doi: 10.1002/ijc.27485, PMID: 22323075

[21] Pretet JL Jacquard AC Carcopino X Charlot JF Bouhour D Kantelip B. Human papillomavirus (HPV) genotype distribution in invasive cervical cancers in France: EDITH study. Int J Cancer. (2008) 122:428–32. doi: 10.1002/ijc.23092, PMID: 17893882

[22] Gallegos-Bolanos J Rivera-Dominguez JA Presno-Bernal JM Cervantes-Villagrana RD. High prevalence of co-infection between human papillomavirus (HPV) 51 and 52 in Mexican population. BMC Cancer. (2017) 17:531. doi: 10.1186/s12885-017-3519-7, PMID: 28789619 PMC5549346

[23] Chan PK Ho WC Chan MC Wong MC Yeung AC Chor JS. Meta-analysis on prevalence and attribution of human papillomavirus types 52 and 58 in cervical neoplasia worldwide. PLoS One. (2014) 9:e107573. doi: 10.1371/journal.pone.0107573, PMID: 25229350 PMC4168000

[24] Zhao S Zhao X Hu S Lu J Duan X Zhang X. Distribution of high-risk human papillomavirus genotype prevalence and attribution to cervical precancerous lesions in rural North China. Chin J Cancer Res. (2019) 31:663–72. doi: 10.21147/j.issn.1000-9604.2019.04.10, PMID: 31564809 PMC6736663

[25] Zhang C Park JS Grce M Hibbitts S Palefsky JM Konno R. Geographical distribution and risk association of human papillomavirus genotype 52-variant lineages. J Infect Dis. (2014) 210:1600–4. doi: 10.1093/infdis/jiu310, PMID: 24879800 PMC4539913

[26] Choi YJ Ki EY Zhang C Ho WC Lee SJ Jeong MJ. Analysis of sequence variation and risk Association of Human Papillomavirus 52 variants circulating in Korea. PLoS One. (2016) 11:e0168178. doi: 10.1371/journal.pone.0168178, PMID: 27977741 PMC5158036

[27] Gong Y Wang Y Zhou Q Qu W Chen F Wang Y. The possible impact of novel mutations in human papillomavirus 52 on the infection characteristics. Microb Genom. (2023) 9:mgen000962. doi: 10.1099/mgen.0.000962 37103992 PMC10210937

[28] Guo F Hirth JM Berenson AB. Comparison of HPV prevalence between HPV-vaccinated and non-vaccinated young adult women (20-26 years). Hum Vaccin Immunother. (2015) 11:2337–44. doi: 10.1080/21645515.2015.1066948, PMID: 26376014 PMC4635939

[29] Luostarinen T Af Geijersstam V Bjorge T Eklund C Hakama M Hakulinen T. No excess risk of cervical carcinoma among women seropositive for both HPV16 and HPV6/11. Int J Cancer. (1999) 80:818–22. doi: 10.1002/(SICI)1097-0215(19990315)80:6<818::AID-IJC4>3.0.CO;2-T, PMID: 10074912

[30] Chaturvedi AK Katki HA Hildesheim A Rodriguez AC Quint W Schiffman M. Human papillomavirus infection with multiple types: pattern of coinfection and risk of cervical disease. J Infect Dis. (2011) 203:910–20. doi: 10.1093/infdis/jiq139, PMID: 21402543 PMC3068034

[31] Trottier H Mahmud S Costa MC Sobrinho JP Duarte-Franco E Rohan TE. Human papillomavirus infections with multiple types and risk of cervical neoplasia. Cancer Epidemiol Biomark Prev. (2006) 15:1274–80. doi: 10.1158/1055-9965.EPI-06-0129 16835323

[32] Iacobone AD Bottari F Radice D Preti EP Franchi D Vidal Urbinati AM. Distribution of high-risk human papillomavirus genotypes and multiple infections in Preneoplastic and neoplastic cervical lesions of unvaccinated women: a cross-sectional study. J Low Genit Tract Dis. (2019) 23:259–64. doi: 10.1097/LGT.0000000000000487, PMID: 31592973

[33] Salazar KL Zhou HS Xu J Peterson LE Schwartz MR Mody DR. Multiple human papilloma virus infections and their impact on the development of high-risk cervical lesions. Acta Cytol. (2015) 59:391–8. doi: 10.1159/000442512, PMID: 26674365

[34] Wu P Xiong H Yang M Li L Wu P Lazare C. Co-infections of HPV16/18 with other high-risk HPV types and the risk of cervical carcinogenesis: a large population-based study. Gynecol Oncol. (2019) 155:436–43. doi: 10.1016/j.ygyno.2019.10.003, PMID: 31604662

[35] Carrillo-Garcia A Ponce-de-Leon-Rosales S Cantu-de-Leon D Fragoso-Ontiveros V Martinez-Ramirez I Orozco-Colin A. Impact of human papillomavirus coinfections on the risk of high-grade squamous intraepithelial lesion and cervical cancer. Gynecol Oncol. (2014) 134:534–9. doi: 10.1016/j.ygyno.2014.06.018, PMID: 24979052

[36] Spinillo A Dal Bello B Alberizzi P Cesari S Gardella B Roccio M. Clustering patterns of human papillomavirus genotypes in multiple infections. Virus Res. (2009) 142:154–9. doi: 10.1016/j.virusres.2009.02.004, PMID: 19428748

[37] Dahlstrom LA Ylitalo N Sundstrom K Palmgren J Ploner A Eloranta S. Prospective study of human papillomavirus and risk of cervical adenocarcinoma. Int J Cancer. (2010) 127:1923–30. doi: 10.1002/ijc.25408, PMID: 20473898 PMC2930102

[38] Bosch FX Burchell AN Schiffman M Giuliano AR de Sanjose S Bruni L. Epidemiology and natural history of human papillomavirus infections and type-specific implications in cervical neoplasia. Vaccine. (2008) 26:K1–K16. doi: 10.1016/j.vaccine.2008.05.064, PMID: 18847553

[39] So KA Lee IH Lee KH Hong SR Kim YJ Seo HH. Human papillomavirus genotype-specific risk in cervical carcinogenesis. J Gynecol Oncol. (2019) 30:e52. doi: 10.3802/jgo.2019.30.e52 31074234 PMC6543103

[40] Correa RM Baena A Valls J Colucci MC Mendoza L Rol M. Distribution of human papillomavirus genotypes by severity of cervical lesions in HPV screened positive women from the ESTAMPA study in Latin America. PLoS One. (2022) 17:e0272205. doi: 10.1371/journal.pone.0272205, PMID: 35905130 PMC9337688

[41] Manawapat-Klopfer A Wang L Haedicke-Jarboui J Stubenrauch F Munk C Thomsen LT. HPV16 viral load and physical state measurement as a potential immediate triage strategy for HR-HPV-infected women: a study in 644 women with single HPV16 infections. Am J Cancer Res. (2018) 8:715–22. PMID: 29736316 PMC5934561

[42] Zhao X Zhao S Hu S Zhao K Zhang Q Zhang X. Role of human papillomavirus DNA load in predicting the long-term risk of cervical Cancer: a 15-year prospective cohort study in China. J Infect Dis. (2019) 219:215–22. doi: 10.1093/infdis/jiy507, PMID: 31067317

[43] Baumann A Henriques J Selmani Z Meurisse A Lepiller Q Vernerey D. HPV16 load is a potential biomarker to predict risk of high-grade cervical lesions in high-risk HPV-infected women: a large longitudinal French hospital-based cohort study. Cancers (Basel). (2021) 13:4149. doi: 10.3390/cancers13164149, PMID: 34439304 PMC8394477

[44] Hortlund M van Mol T Van de Pol F Bogers J Dillner J. Human papillomavirus load and genotype analysis improves the prediction of invasive cervical cancer. Int J Cancer. (2021) 149:684–91. doi: 10.1002/ijc.33519, PMID: 33586149

[45] Sakamoto J Kamiura S Okayama K Okodo M Shibata T Osaka Y. Single type infection of human papillomavirus as a cause for high-grade cervical intraepithelial neoplasia and invasive cancer in Japan. Papillomavirus Res. (2018) 6:46–51. doi: 10.1016/j.pvr.2018.10.001, PMID: 30401640 PMC6222286

[46] Aro K Nieminen P Louvanto K Jakobsson M Virtanen S Lehtinen M. Age-specific HPV type distribution in high-grade cervical disease in screened and unvaccinated women. Gynecol Oncol. (2019) 154:354–9. doi: 10.1016/j.ygyno.2019.05.024, PMID: 31176553

[47] Huică I Iancu IV Botezatu A Pleşa A Socolov D Teleman S. Factors associated with persistence of HPV genital infection in a small cohort of Romanian women. Acta Clin Croat. (2019) 58:410–6. doi: 10.20471/acc.2019.58.03.02 31969751 PMC6971790

[48] Sammarco ML Del Riccio I Tamburro M Grasso GM Ripabelli G. Type-specific persistence and associated risk factors of human papillomavirus infections in women living in Central Italy. Eur J Obstet Gynecol Reprod Biol. (2013) 168:222–6. doi: 10.1016/j.ejogrb.2013.01.012, PMID: 23395560

[49] Denis F Hanz S Alain S. Clairance, persistance et récidive de l'infection à Papillomavirus [Clearance, persistence and recurrence of HPV infection]. Gynecol Obstet Fertil. (2008) 36:430–40. doi: 10.1016/j.gyobfe.2008.02.008 18417407

[50] Song F Yan P Huang X Wang C Du H Qu X. Roles of extended human papillomavirus genotyping and multiple infections in early detection of cervical precancer and cancer and HPV vaccination. BMC Cancer. (2022) 22:42. doi: 10.1186/s12885-021-09126-3, PMID: 34991494 PMC8734293

[51] Hequet D Rouzier R. Determinants of geographic inequalities in HPV vaccination in the most populated region of France. PLoS One. (2017) 12:e0172906. doi: 10.1371/journal.pone.0172906, PMID: 28257434 PMC5336257

