# Supplementary material for: Relative distribution of HPV genotypes in histological cervical samples and associated grade lesion in a women population over the last 16 years in Burgundy, France
Source: Front Med (Lausanne). 2023 Aug 11;10:1224400. doi: 10.3389/fmed.2023.1224400 (PMC10453809; doi:10.3389/fmed.2023.1224400)
Supplement: Supplementary file 1 [file Table_1.docx]

**Table S1 Interactions among HPV genotypes in multi-infected women.**

The first column indicates the studied genotype. The 13 following columns, one for each genotype, show the observed coinfections for each genotype listed in the first column. Results are expressed as a percentage of co-infection for the studied genotype of the first column. Per example: row 16, column 31 shows that 11.4 % of the coinfections involving HPV16 contains HPV31. The green boxes indicates percentage over 25%,

When the number of coinfection was below n=15, genotype does not appear in the table.
