# Supplementary figures and images for: Relative distribution of HPV genotypes in histological cervical samples and associated grade lesion in a women population over the last 16 years in Burgundy, France
Source: Front Med (Lausanne). 2023 Aug 11;10:1224400. doi: 10.3389/fmed.2023.1224400 (PMC10453809; doi:10.3389/fmed.2023.1224400)

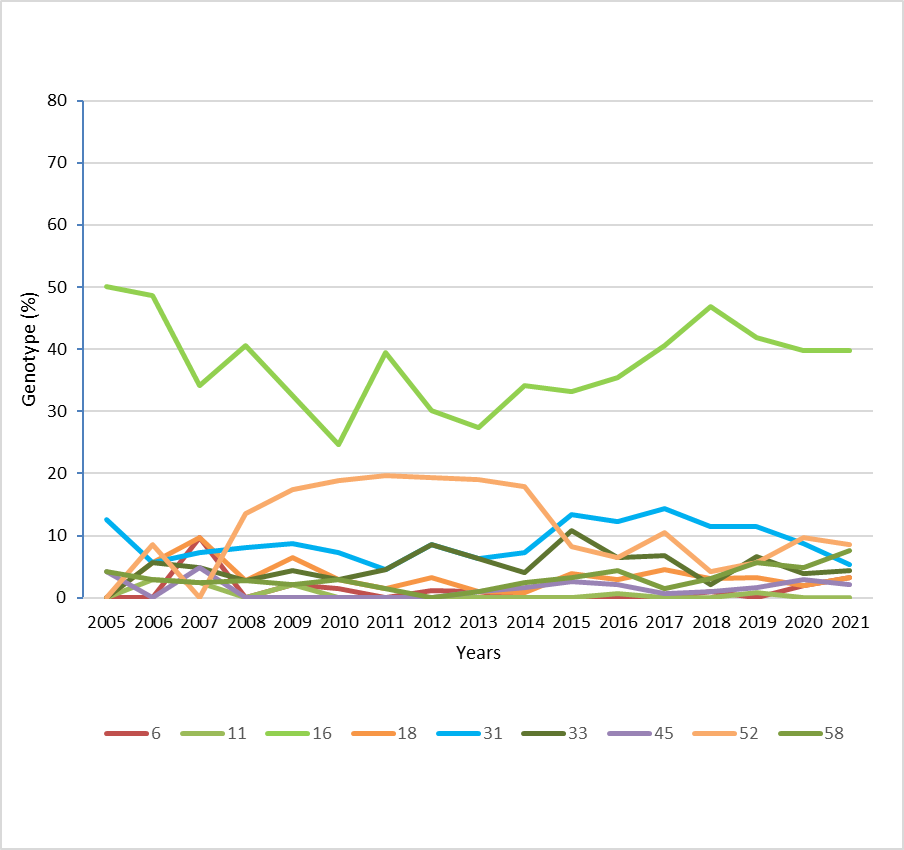


**Fig. S1 Fluctuation of HPV distribution for genotypes targeted by Gardasil 9 vaccine**

Supplement: Supplementary file 2 [file Data_Sheet_1.docx]
